# Brillouin Light Scattering Characterisation of Gray Tone 3D Printed Isotropic Materials

**DOI:** 10.3390/ma15124070

**Published:** 2022-06-08

**Authors:** Fehima Ugarak, Gwenn Ulliac, Julio Andrés Iglesias Martínez, Johnny Moughames, Vincent Laude, Muamer Kadic, Alexis Mosset

**Affiliations:** Institute FEMTO-ST, CNRS, University Bourgogne Franche-Comté, 25000 Besançon, France; ugarakfehima@outlook.com (F.U.); julio.iglesias@femto-st.fr (J.A.I.M.); johnny.moughames@femto-st.fr (J.M.); vincent.laude@femto-st.fr (V.L.)

**Keywords:** 3D printed materials, Brillouin light scattering, elastic properties of polymers

## Abstract

Three-dimensional direct laser writing technology enables one to print polymer microstructures whose size varies from a few hundred nanometers to a few millimeters. It has been shown that, by tuning the laser power during writing, one can adjust continuously the optical and elastic properties with the same base material. This process is referred to as gray-tone lithography. In this paper, we characterize by Brillouin light scattering the complex elastic constant $C_{11}$ of different reticulated isotropic polymers, at longitudinal phonon frequencies of the order of 16 GHz. We estimate the real part of the $C_{11}$ constant to vary from 7 to 11 GPa as a function of laser power, whereas its imaginary part varies between 0.25 and 0.6 GPa. The linear elastic properties are further measured at a fixed laser power as a function of temperature, from $20{\;}^{\circ }$C to $80{\;}^{\circ }$C. Overall, we show that our 3D printed samples have a good elastic quality with high Q factors only ten times smaller than fused silica at hypersonic frequencies.

## 1. Introduction

Three-dimensional direct laser writing has met an exponential rise over the last decade [1,2]. In the early age, its use was mainly directed toward electromagnetic applications and it thus was the object of a quest for the highest resolution [1] and the lowest optical losses [3]. This new technology opens the way to new optical metamaterials as simple as 3D dielectrics [4,5,6,7] or to metals by using the printed sample as a mold [8]. Additional efforts have been made to convert dielectrics to conducting parts via atomic layer deposition [9] through simple thermal evaporation. More recently, 3D direct laser scanning was used for gray tone lithography [10,11]. Indeed, when changing the laser power (LP), the scanning velocity or the hatching/slicing distances, one can obviously impart different degrees of polymerisation and cross-linking during the printing process [11]. All parameters are important and the resulting properties can be more or less sensitive to them. It should be noted that the variations of electromagnetic properties such as the refractive index are rather small [12]. However, even such a small but tunable change is very useful and advantageous for manufacturing optical waveguides [6]. Micro-robotics, acoustics and elasticity have also taken advantage of 3D printing technologies for the manufacture of metamaterials via gray tone lithography [10]. One important example is the implementation of the so-called bi-material beams where one takes advantage of the very sensitive differential deformation between two rather similar materials [10,13].

A few extensive studies have been performed on different polymers to obtain the printable properties as a function of printing parameters, via Raman scattering or other characterisation techniques [14,15]. However, from one setup to another and for different reasons, characterisation must be repeated and adapted each time. In this paper, we aim at characterising via Brillouin light scattering two transparent photoresins called IP-Dip2 and IP-S that are commercially available from the Nanoscribe company [16]. Due to the isotropic elastic behavior of those photoresins and the fact that only longitudinal acoustic phonons can be probed, we limit our analysis to the dependence of the $C_{11}$ elastic constant as a function of writing laser power, writing speed and temperature. We show how the elastic constant can be tuned and to some degree in which range of parameters one should play to obtain the largest changes. All characterisations have been done using the Brillouin Light Scattering (BLS) technique using a multipass Fabry–Pérot interferometer. Ref. [17] Hence, the information we provide regarding both the real part (determining the longitudinal phonon velocity) and the imaginary part (determining the phonon loss) of $C_{11}$ at GHz frequencies is novel and has implications for ultrasonic MEMS applications.

## 2. Fabrication

In this study, we use a commercial 3D micro-printer (Photonic Professional GT+, Nanoscribe GmbH) based on two-photon absorption lithography. The photoresins investigated in this study are the commercial negative tone IP-Dip2 and IP-S (Nanoscribe GmbH) that were customized by Nanoscribe for high resolution and mesoscale applications.

**Figure 1:**
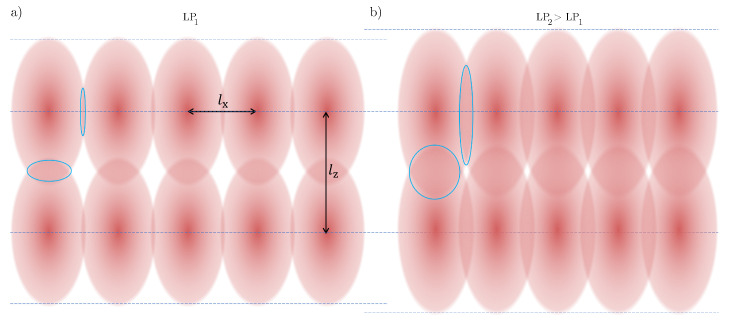
Principle of Direct Laser Writing voxels for different laser power. (**a**) definition of a voxel that is used in our printing and of the hatching distance $l_{x}$ and slicing distance $l_{z}$; (**b**) schematic representation of the voxels for a laser power larger than in (**a**). A larger voxel is obtained, which, for the same slicing, results in a larger overlap of adjacent voxels (overlaps are depicted with blue ellipses).

**Figure 2:**
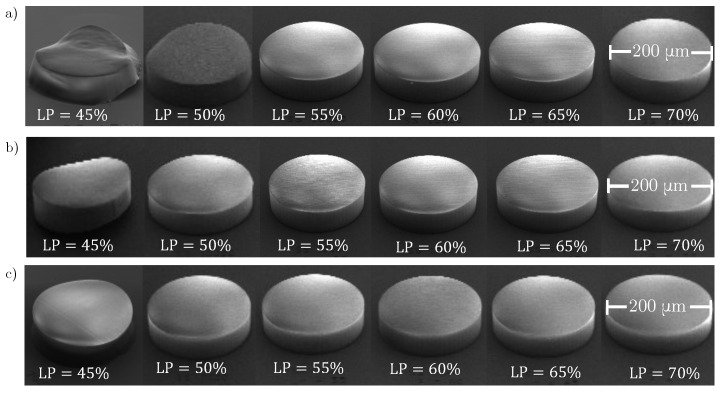
SEM images of IP-Dip2 samples for different laser powers and at different scanning velocities. (**a**) $v_{s}=15$ mm/s; (**b**) $v_{s}=10$ mm/s; (**c**) $v_{s}=7$ mm/s.

**Figure 3:**
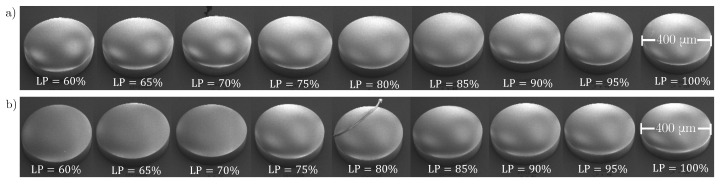
SEM images of IP-S samples for different laser powers and at different scanning velocities. (**a**) $v_{s}=200$ mm/s; (**b**) $v_{s}=100$ mm/s.

We fixed the slicing and hatching distances for each resin. For the IP-Dip2 sample, they were set to $l_{x}=0.3$ μm and $l_{z}=0.2$ μm. For IP-S, we consistently use $l_{x}=1$ μm and $l_{z}=0.5$ μm. As illustrated in Figure 1, it is expected that, for the same hatching and slicing distances, a higher laser power results in a larger overlap between voxels and consequently in an enhancement of the polymerisation of photoresins. To prepare samples, a drop of IP-Dip2 (IP-S) photoresin was deposited on a fused silica (ITO-coated soda lime glass) substrate and photopolymerized using a 63X or 25X microscope objective and a femtosecond laser operating at λ=780 nm. The reference printing parameters are a laser power of 60% and a galvanometric scanning speed of 10 mm/s for IP-Dip2 and 100% and 100 mm/s for IP-S. After printing, the samples were developed for 20 min in a propylene glycol methyl ether acetate (PGMEA) solution to remove the unexposed photoresin and rinsed for 3 min in isopropyl alcohol (IPA) to clear the developer. The printed samples are cylinders with dimensions of [200×200×50] $\mu m^{3}$ and [400×400×50] $\mu m^{3}$ for IP-Dip2 and IP-S, respectively, and the distance between each cylinder was set to 500 μm. This inter-cylinder distance, for both IP-S and IP-Dip2, ensures that the modification of the phonon spectrum is achieved in each cylinder. As the core study, we consider the influence of selected fabrication parameters on the elastic properties of the printed materials. Figure 2 and Figure 3 show SEM images of samples that are used for measurements. Overall, they are homogeneous in both shape and aspect. The minor blemishes and their domed tops do not affect measurements of bulk longitudinal phonons by BLS and thus the determination of the elastic constant $C_{11}$.

## 3. Fundamentals of BLS for Isotropic Media

As stated in the Introduction, the BLS contact-less characterisation technique is used in order to estimate the effective elastic constant $C_{11}$ of the samples. We remind the reader that, in our study, we only consider linear elasticity. Thus, the stress tensor σ can be related to the strain tensor ϵ by the 4th rank elasticity tensor *C* as:

(1) $$\sigma_{ij}=C_{ijkl}\epsilon_{kl}.$$

Taking into account major and minor symmetry of the tensor, the elasticity tensor can be simplified drastically to have only 21 independent elasticity terms. However, if, in addition, we consider the material as isotropic, the simplification is even more drastic and only two independent parameters are left. It is common to express the new elasticity tensor in Voigt notation:

(2) $$C_{\alpha \beta }=\left[\begin{array}{cccccc} C_{11} & C_{12} & C_{12} & 0 & 0 & 0\\ C_{12} & C_{11} & C_{12} & 0 & 0 & 0\\ C_{12} & C_{12} & C_{11} & 0 & 0 & 0\\ 0 & 0 & 0 & C_{44} & 0 & 0\\ 0 & 0 & 0 & 0 & C_{44} & 0\\ 0 & 0 & 0 & 0 & 0 & C_{44} \end{array}\right].$$

with $C_{44}$ being the shear modulus and the relation $C_{12}=C_{11}-2C_{44}$. In Voigt notation, the indices α=(ij) and β=(kl) replace pairs of symmetric indices. As our material is isotropic, both elastic coefficients can be expressed by engineering moduli as follows: $C_{11}=B+4G/3$ and $C_{44}=G$, where *B* and *G* are the bulk and shear moduli, respectively. Another common parametrization for isotropic materials uses Young’s modulus *E* and Poisson’s ratio η, with relations $C_{11}=E\left(1-\eta \right)/\left[\left(1+\eta \right)\left(1-2\eta \right)\right]$ and $C_{44}=E/\left[2\left(1+\eta \right)\right]$.

BLS relies on the inelastic scattering of light by spontaneous thermal fluctuations in a material. Those fluctuations correspond to acoustic phonons. Scattering of light in directions other than the incident one is caused by fluctuations of the material’s dielectric tensor and depends both on the optical properties of the material and on the light penetration into the sample [18]. In the scattered light, there are in principle contributions from bulk phonons, through a volumetric opto-elastic mechanism, and from surface phonons, through a surface-ripple mechanism. Which of those two mechanisms is dominant in the BLS spectrum depends on the sample’s thickness and on the optical properties of the material, such as its transparency at the given excitation wavelength. With our samples, bulk phonons are dominant and will only be considered in the following.

**Figure 4:**
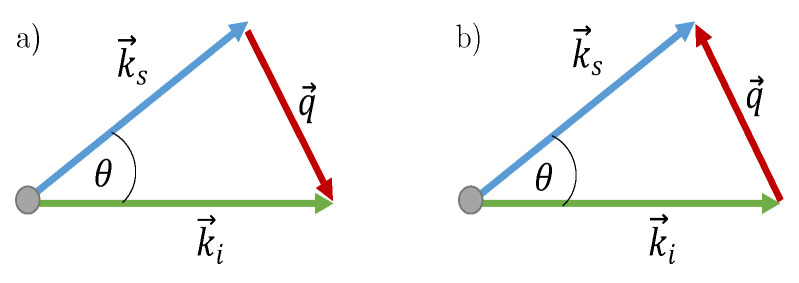
Vector representation of the light-scattering processes via bulk phonons (**a**) Stokes process; (**b**) Anti-Stokes process.

Figure 4 illustrates the general light-scattering processes involving bulk phonons in a Stokes and anti-Stokes processes. In those processes, the law of conservation of momentum is given by

(3) $$\hbar {\vec{k}}_{s}-\hbar {\vec{k}}_{i}=\pm \hbar \vec{q}$$

and the conservation of energy is given by

(4) $$\hbar \omega_{s}-\hbar \omega_{i}=\pm \hbar \omega_{q}.$$

Here, **${\vec{k}}_{i}$** and **${\vec{k}}_{s}$** are the incident and scattered wavevectors, and $\omega_{i}$ and $\omega_{s}$ are the angular frequencies of inci dent and scattered light, respectively. The positive and negative signs on the right-hand sides correspond to ’anti-Stokes’ and ’Stokes’ processes, respectively. The anti-Stokes process is a process of annihilation (or absorption) of a phonon, whereas the Stokes process represents a creation (or emission) of a phonon during scattering. Angle θ between **${\vec{k}}_{i}$** and **${\vec{k}}_{s}$** is the scattering angle that depends on the scattering geometry [19]. Our measurements were performed in the back-scattering geometry where the angle θ=π. This backscattering arrangement provides a comparably larger interaction volume, allowing for a higher temporal resolution and Brillouin spectra that can be recorded faster, compared to other common scattering geometries [20]. The connection between the frequency shift *f* of Brillouin peaks and the phase velocity $v_{L}$ of the longitudinal bulk acoustic phonon is

(5) $$v_{L}=\frac{2\pi f}{q_{L}}=\frac{\lambda f}{2n}\sin\left(\frac{\theta }{2}\right)$$

where **${\vec{q}}_{L}=2n{\vec{k}}_{i}$** is the phonon wavevector with **$∥{\vec{k}}_{i}∥=2\pi /\lambda$**, where λ is the wavelength of the incident light. From the measurement of the frequency of the longitudinal bulk acoustic phonons, the linewidth $\Gamma_{b}$ (FWHM) of the Brillouin peak, and the mass density of the material, one can estimate the real and imaginary parts of the longitudinal modulus $C_{11}$ [19]. The real part of the longitudinal modulus is obtained through the relation

(6) $$ℜ\left(C_{11}\right)=\rho v_{L}^{2}=\frac{\rho \lambda^{2}}{4n^{2}}f^{2}.$$

Imaginary part of the longitudinal modulus $ℑc_{11}$ depends on the linewidth $\Gamma_{b}$ (FWHM) of the Brillouin peak through

(7) $$ℑ\left(C_{11}\right)=\frac{\rho \lambda^{2}}{4n^{2}}f\frac{\Gamma_{b}}{2\pi }.$$

**Figure 5:**
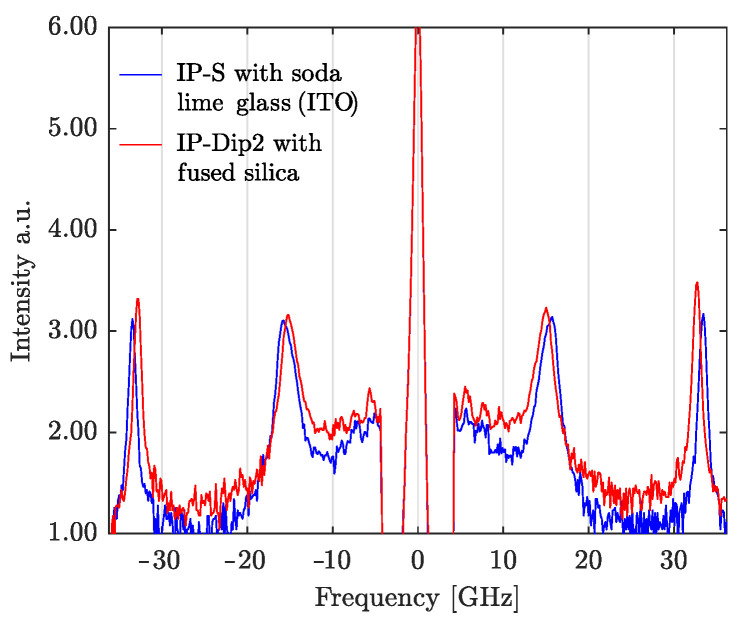
Example of BLS spectra measured directly from the setup for two different photoresins on two different substrates. Clear anti-Stokes and Stokes peaks are observed in both cases.

As an illustration, Figure 5 shows a typical BLS recorded spectrum. The central peak is the Rayleigh elastic scattering of the probe laser. The Stokes and anti-Stokes frequency shifts are visible respectively to the left and to the right side of the central peak. The peaks around ±33 GHz are induced by the longitudinal bulk phonon of the substrate. Peaks for photoresins appear around ±16 GHz.

## 4. Experimental Setup

**Figure 6:**
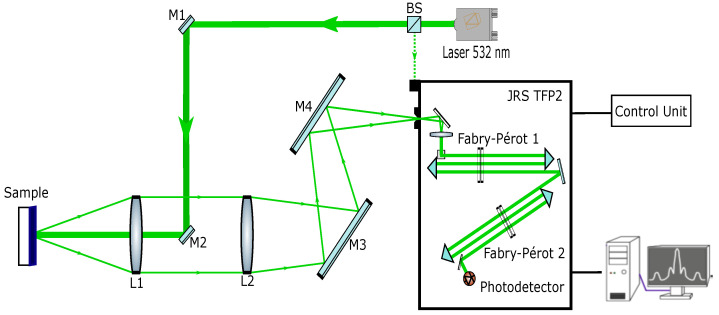
Brillouin light back-scattering setup.

Figure 6 presents the main components of our experimental setup. We use a continuous single longitudinal mode Nd-YAG laser, doubled at 532 nm, vertically polarized, to illuminate the sample with an incident power of 20 mW. A small amount of the laser beam (∼1%) is diverted by a beam splitter (BS) in order to obtain a reference beam (green dashed line), giving the Rayleigh elastic peak. The incident laser beam is controlled by mirrors M1 and M2. Lens L1 (f=100 mm, N.A. =0.1) focuses the incident laser beam, giving a spot diameter smaller than 50 μm, and collects backscattered light. Mirror M2 is very small compared to the area of the pupil of lens L1, in order to reduce the losses of collected scattering light in comparison with a standard 50/50 beam splitter. We illuminate the sample under quasi-normal incidence (∼$2^{\circ }$ with respect to the sample surface) to avoid dazzling the detector with reflected light. We assume that this small angular detuning does not affect the Brillouin frequency shift because the wavenumber of bulk acoustic phonons in isotropic media is independent of the incidence angle with respect to the normal to the surface. Backscattered light is focused by achromatic lens L2 (f=400 mm) in a Sandercock-type 3+3-pass tandem Fabry–Pérot interferometer (TFP-2 HC) with respect to its numerical aperture. The two steering mirrors M3 and M4 can be adjusted so the backscattered light beam enters the entrance pinhole of the interferometer. The very weak signal produced by spontaneous inelastic scattering of light by bulk acoustic phonons is filtered with a high contrast ratio and detected by a synchronized photomultiplier. In order to observe and position the laser spot onto the sample, it was inevitable to install a removable confocal-like setup with a beam splitter, a white light source and a CMOS camera, which are not represented here. Furthermore, to perform measurements as a function of the temperature of the sample, the sample was inserted inside a Linkam head (not represented) with a thin window transparent to visible light. The temperature change was performed from $30{\;}^{\circ }$C to $80{\;}^{\circ }$C in steps of $10{\;}^{\circ }$C and back.

## 5. Results for Isotropic Materials IP-Dip2 and IP-S

In this section, we analyze the dependence of the complex-valued elastic constant $C_{11}$ for different writing laser powers and scanning velocities $v_{s}$. Both IP-S and IP-Dip2 samples are considered. In order to retrieve the elastic properties, we assume that IP-S is a typical amorphous polymer with mass density ρ=1280 kg/$m^{3}$ that, at room temperature (T=293 K), has a refractive index n=1.52 [21]. IP-Dip2 is a photoresin with a refractive index n=1.55 [21] at room temperature and mass density ρ=1170 kg/$m^{3}$.

As explained before, one can in principle change the effective properties of the printed material via at least three different controls: the laser power, the hatching/slicing distance and the scanning velocity. In this study, we keep the hatching/slicing distances constant since these parameters are crucial for maintaining low surface roughness as well as high writing resolution.

**Figure 7:**
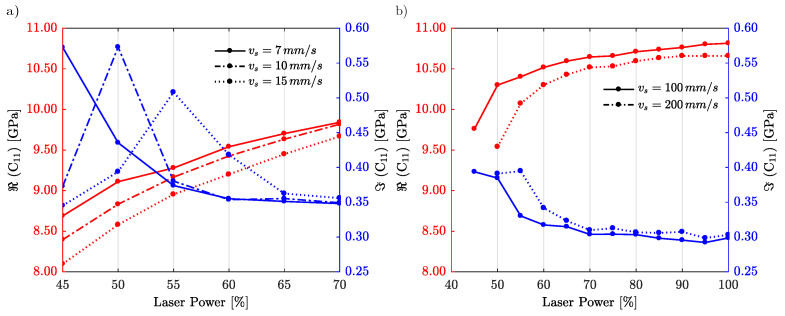
Measured elastic constant $c_{11}$ for different writing laser powers, and for (**a**) IP-Dip2 and (**b**) IP-S. The red color represents the real part of the longitudinal modulus and blue represents its imaginary part. Each type of line corresponds to a different scanning velocity.

In Figure 7, we show the measured elastic constant $C_{11}$ for IP-Dip2 (panel (a)) and for IP-S (panel (b)). The study is done for both photoresins as a function of the relative laser power. In both cases, we show results for different scanning velocities $v_{s}$. For IP-Dip2, we limit the laser power to LP=70% in order to avoid burning the photoresin. For both photoresins, we observe a stiffening process as a function of the laser power. As the laser power increases, the value of the real part of the longitudinal modulus $C_{11}$ increases before reaching a maximum. Simultaneously, the imaginary part of $C_{11}$ decreases to reach a minimum. Hence, viscoelastic damping reduces with the laser power.

When the writing velocity increases, the effective dose received by each voxel becomes smaller and polymerisation via diffusion decreases, resulting in a softer material (lower elastic constant). We attribute this effect to the enhancement of the cross-linking and to the fact that two-photon absorption induces a saturation of the polymerisation process [11]. For IP-S, this aspect is more pronounced and we reach a plateau very fast. It is often observed in the literature that faster polymerisation combined with better diffusion results in more homogeneous sample properties and thus in a better surface and volume quality [14]. Here, we furthermore emphasize the importance of damping that increases for both photoresins when the laser power decreases. As observed in previous studies, for low laser power, polymerisation can in principle be rather inhomogeneous and some parts can contain monomers (not polymerised). However, we also note a decrease in damping in the case of IP-Dip2 for the lowest laser power. This surprising phenomenon was checked with additional samples and repeating measurements; results have always been consistent. We attribute this effect to a process of grain-like formation in the samples when the quality deteriorates [22]. This deterioration of quality is often attributed to domain formation.

Interestingly, we observed that in general for both photoresins and for all printing conditions the imaginary part is rather low and thus bulk longitudinal phonons can propagate in these polymers in the GHz regime with reasonable loss. The quality factor times frequency product, Qf, which is expected to be a constant in the ultrasonic range for most viscoelastic materials, is at best of the order of $50\;10^{10}$Hz, only smaller by a factor 10 compared to Qf measured for fused silica optical fibers [23].

The datasheet for the IP-S resin indicates that E=5 GPa from static measurements (at very low mechanical frequencies) [16]. In the MHz range, Young’s modulus can be estimated from the analysis of the resonances of beams. The reported value for Young’s modulus is E=10 GPa for the IP-S photoresin for frequencies between 1 and 3 MHz [7]. For example, converting to elastic constants, this yields to $Re\left(c_{11}\right)=E\left(1-\nu \right)/\left[\left(1+\nu \right)\left(1-2\nu \right)\right]\approx 15$ GPa assuming a Poisson’s ratio ν=0.3. Hence, we observe similar values in the GHz regime.

## 6. Temperature Dependence of Elastic Constants

**Figure 8:**
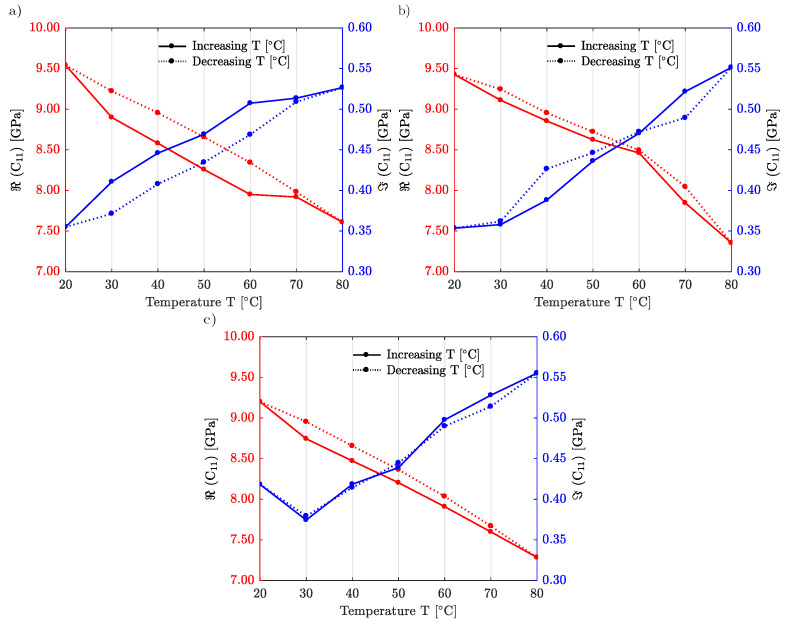
Temperature dependence of the elastic constant $C_{11}$ for IP-Dip2. The scanning speed is (**a**) $v_{s}=7$ mm/s; (**b**) $v_{s}=10$ mm/s; and (**c**) $v_{s}=15$ mm/s.

Lastly, we examine the dependence of elastic properties with temperature. For that purpose, we insert the samples into a Linkam chamber installed on the sample holder to control its temperature. Figure 8 shows the temperature dependence of the elastic constant $C_{11}$ for IP-Dip2 and for a single laser power of LP=60%, but for three different scanning velocities, $v_{s}=7$ mm/s in panel (a), $v_{s}=10$ mm/s in panel (b), and $v_{s}=15$ mm/s in panel (c). We first note that the temperature dependence is rather linear and that the initial properties are recovered for all samples after a full cycle. For the lowest scanning velocity, a small difference appears between the heating cycle and the cooling cycle. As expected for many polymers, as temperature increases, the material becomes softer: the real part of the longitudinal modulus decreases whereas the imaginary part increases, as often for viscous media. However, we note that the imaginary part is mostly two orders of magnitude smaller than the real part of $C_{11}$ and that the polymer is indeed in a very stiff and limited lossy state, making it suitable for acoustic experiments without detrimental absorption over the full range of temperature study.

**Figure 9:**
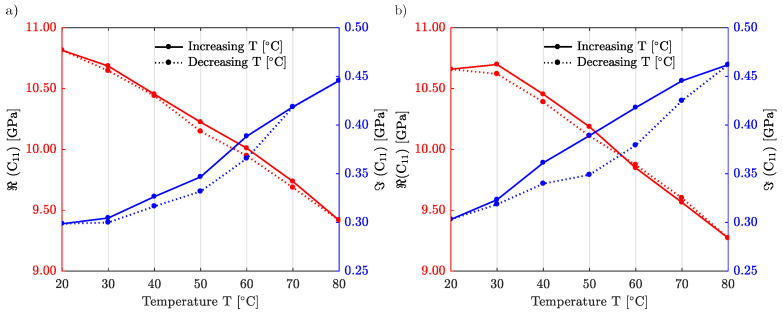
Temperature dependence of the elastic constant $C_{11}$ for IP-S. The scanning velocity is (**a**) $v_{s}=100$ mm/s, (**b**) $v_{s}=200$ mm/s.

Figure 9 proposes a similar study but for IP-S. The laser power is set to LP=100%. Two different scanning velocities are considered, $v_{s}=100$ mm/s in panel (a) and $v_{s}=200$ mm/s in panel (b). The hysteresis deviation between increasing and decreasing temperature cycles is now quite small. IP-S is thus more stable and less sensitive to ambient temperature variations as compared to IP-Dip2.

## 7. Conclusions

In this paper, we have characterized using Brillouin light scattering the elastic properties of two commercially available photoresins used for two-photon lithography. Specifically, the Brillouin shift of longitudinal phonons was recorded as a function of writing laser power, scanning velocity, and sample temperature. Measurements show that the real part of the elastic constant $C_{11}$ changes relatively by about 10 to 15%, whereas the imaginary part changes by about the same amount but in the opposite direction. The elastic constant can thus be tuned continuously between 7 and 11 GPa at phonon frequencies of the order of 16 GHz. Concurrently, viscoelastic loss can be tuned between 0.25 and 0.6 GPa. We hope that our findings can be useful for the design of smart structures by gray-tone lithography and can motivate the use of 3D printing at the microscale for acoustics in the GHz regime.

## Data Availability

All data are available upon reasonable request from the corresponding authors.

## Sample Availability

Samples are available from the authors.
